# External Validation of the Charlson Comorbidity Index-based Model for Survival Prediction in Thai Patients Diagnosed with Dementia

**DOI:** 10.1186/s12877-024-05238-0

**Published:** 2024-08-12

**Authors:** Nida Buawangpong, Phichayut Phinyo, Chaisiri Angkurawaranon, Atiwat Soontornpun, Wichuda Jiraporncharoen, Wachiranun Sirikul, Kanokporn Pinyopornpanish

**Affiliations:** 1 Department of Family Medicine, Faculty of Medicine, Chiang Mai University, 110 Inthawarorot Rd., Chiang Mai, 50200 Thailand; 2 Global Health and Chronic Conditions Research Group, Chiang Mai University, Chiang Mai, 50200 Thailand; 3 Center for Clinical Epidemiology and Clinical Statistics, Faculty of Medicine, Chiang Mai University, Chiang Mai, 50200 Thailand; 4 Musculoskeletal Science and Translational Research (MSTR), Chiang Mai University, Chiang Mai, 50200 Thailand; 5 Division of Neurology, Department of Internal Medicine, Faculty of Medicine, Chiang Mai University, Chiang Mai, 50200 Thailand; 6 Department of Community Medicine, Faculty of Medicine, Chiang Mai University, Chiang Mai, 50200 Thailand

**Keywords:** Dementia, Alzheimer’s disease, Comorbidity, Survival time

## Abstract

**Background:**

The Charlson Comorbidity Index (CCI) is commonly employed for predicting mortality. Nonetheless, its performance has rarely been evaluated in patients with dementia. This study aimed to examine the predictive capability of the CCI-based model for survival prediction in Thai patients diagnosed with dementia.

**Methods:**

An external validation study was conducted using retrospective data from adults with dementia who had visited the outpatient departments at Maharaj Nakorn Chiang Mai Hospital between 2006 and 2012. The data obtained from electronic medical records included age, gender, date of dementia diagnosis and death, types of dementia, and comorbidities at the time of dementia diagnosis. The discriminative ability and calibration of the CCI-based model were estimated using Harrell’s C Discrimination Index and visualized with calibration plot. As the initial performance did not meet satisfaction, model updating and recalibration were performed.

**Results:**

Of 702 patients, 56.9% were female. The mean age at dementia diagnosis was 75.22 (SD 9.75) year-old. During external validation, Harrell's C-statistic of the CCI-based model was 0.58 (95% CI, 0.54–0.61). The model showed poor external calibration. Model updating was subsequently performed. All updated models demonstrated a modest increase in Harrell's C-statistic. Temporal recalibration did not significantly improve the calibration of any of the updated models.

**Conclusion:**

The CCI-based model exhibited fair discriminative ability and poor calibration for predicting survival in Thai patients diagnosed with dementia. Despite attempts at model updating, significant improvements were not achieved. Therefore, it is important to consider the incorporation of other influential prognostic factors.

**Supplementary Information:**

The online version contains supplementary material available at 10.1186/s12877-024-05238-0.

## Background

Many elderly individuals all around the world have been affected by cognitive decline, often known as dementia, which is linked to their increasing age. Dementia is a brain condition marked by a deterioration in cognition across one or more cognitive areas. The impairments represent a reduction in function from baseline and interference with everyday self-care and independence[1]. There are an estimated 44 million people with dementia worldwide, and by 2050 that number is predicted to triple[2]. In Thailand, the prevalence of dementia among older adults ranged from two to ten percent, with the trend predicted to increase with increasing age[3]. Dementia results in an increased financial burden on the health care system and is a leading cause of death[4, 5].

A recent systematic review[6] identified gaps in the literature evaluating the effect of dementia on mortality. Characterizing a dementia prognosis is essential for evaluation and management decisions, communication with patients and their families, and advanced care planning[7, 8]. Many factors have been reported as predictive of mortality in patients with dementia such as patient factors; age, gender, education, ethnicity, characteristics of disease; severity, functional impairment, specific symptoms and signs, and co-morbidity.

Dementia frequently coexists with multiple health conditions[9, 10]. Comorbidities are also prevalent among the elderly population and have been extensively investigated as predictors of mortality in dementia and Alzheimer's disease [11, 12]. There is evidence that the model consisting of patients’ age, gender, body mass index, smoking status, functions, and chronic conditions, including cancer, heart disease, diabetes, and lung disease, could be used for mortality prediction in dementia [13]. A widely used tool for estimating survival probability in individuals with multiple chronic conditions is the Charlson Comorbidity Index (CCI) [14]. CCI is usually utilized to predict ten-year survival in patients with multiple comorbidities including dementia and is a method of categorizing comorbidities of patients based on the International Classification of Diseases (ICD) diagnosis codes found in administrative data, such as hospital electronic medical records. Each comorbidity category has an associated weight (from 1 to 6), based on the adjusted risk of mortality or resource use, and the sum of all the weights results in a single comorbidity score for a patient. A score of zero indicates that no comorbidities were found. The higher the score, the more likely the predicted outcome would result in mortality or higher resource use.

There is a growing need for a larger number of studies to demonstrate the potential utility of CCI in predicting mortality in dementia and to assess its accuracy [15, 16]. In addition, the predictive performance may vary according to the variation in population, race, or ethnicity [17, 18]. Therefore, our study aimed to explore the predictive performance of the CCI-based model for predicting the ten-year survival probability for Thai patients with dementia.

## Methods

### Study Design

A retrospective observational cohort study was performed. The study was reported in accordance with the Transparent Reporting of a multivariable prediction model for Individual Prognosis Or Diagnosis (TRIPOD) statement [19] (Additional file 1).

### Setting

Maharaj Nakorn Chiang Mai Hospital (a university-affiliated, tertiary care center), Chiang Mai, Thailand.

### Study Population

Since the inception of the hospital's electronic medical records in 2006, adult patients diagnosed with dementia who visited the Outpatient Department at Maharaj Nakorn Chiang Mai Hospital between January 2006 to December 2012 (tracking from ICD-10 codes [20] as shown in Additional file 2) were included. This duration allows for a sufficient follow-up period of at least 10 years for the patients. The patients visited an outpatient department at Maharaj Nakorn Chiang Mai Hospital and were diagnosed with any type of dementia by the neurologists according to the Fifth Edition of the Diagnostic and Statistical Manual of Mental Disorders (DSM-5) 2013 [21] and Thai Clinical Practice Guidelines for Dementia^3^. The patients with prior diagnosis of dementia from other hospital settings and no recorded data of diagnosis information, or reversible dementia e.g., infectious process, endocrine disorder, or nutritional deficiency, or incomplete demographic data (history of diagnosed dementia, date of diagnosed dementia, and death date) were excluded.

### Study Size Estimation for External Validation of the Original CCI Score

To estimate the required sample size for the external validation of the original CCI score, the simulation-based approach by Riley et al. [22] was applied. However, there were neither figures nor data available for the distribution of the predicted linear risk scores and concordance statistics in the original CCI article. As suggested by Riley et al., we collected 200 pilot samples for estimating the skewed normal distribution of the linear prediction of the original CCI score. In the original CCI study, baseline survival probability was 98.3%, with no reports of ten-year survival or censor probability. Therefore, the distribution of ten-year survival and censorship probability was estimated from 200 pilot samples, which were 32.0% and 18.3%, respectively. Of the 865 patients diagnosed with dementia who visited the OPD of our hospital between 2006 and 2012, we expected to have at least 700 patients who were eligible for external validation. A simulation of 1000 bootstrapping was used to determine the precision (mean standard error (SE)) of the calibration slope and the number of events. A total of 335 observed events during the ten-year follow-up were required to achieve the target SE of a calibration slope of 0.30.

### Variables and Data Sources

All data was retrieved and extracted from the electronic medical records of Maharaj Nakorn Chiang Mai Hospital. The secondary data of patients’ age, gender, date of dementia diagnosis and death, types of dementia, and comorbidities at the time of dementia diagnosis were assessed. For types of dementia, NINCDS-ADRDA criteria were applied for possible or probable Alzheimer’s disease [23]. NINDS/AIREN criteria were applied for possible vascular dementia [24]. Parkinson’s disease dementia was diagnosed according to clinical diagnostic criteria for dementia associated with Parkinson’s disease [25]. Dementia with Lewy bodies (DLB) was diagnosed according to the Consortium on DLB [26]. Frontotemporal dementia (FTD) diagnostic criteria were determined according to the Work Group on FTD and Pick’s disease [27]. The comorbidities were defined using ICD-10 codes [20] available in the electronic medical records (Additional file 3).

### Determinant

Twenty-one predictors were included within the model, including variables from the CCI. Age was included as a categorical variable with five levels (< 50 years, 50–59 years, 60–69 years, 70–79 years, and ≥ 80 years). The comorbidities were included as binary variables (presence or absence) and consisted of myocardial infarction (MI), congestive heart failure (CHF), peripheral vascular disease (PVD), cerebrovascular accident (CVA) or transient ischemic attack (TIA), chronic obstructive pulmonary disease (COPD), connective tissue disease (CTD), peptic ulcer (PU), hemiplegia, moderate to severe chronic kidney disease (CKD), solid tumor, leukemia, lymphoma, and acquired immunodeficiency syndrome (AIDS). Liver disease was included as a categorical variable with two levels (mild and moderate to severe). Type 2 diabetes mellitus (DM) was included as a categorical variable with two levels (uncomplicated and complicated).

### Outcome

The outcome was all-cause mortality at ten years. All-cause death status and death date were obtained from the Thai civil registration system database. Survival time was calculated as the duration between the date of dementia diagnosis and either the date of death or the last recorded date within the ascertainment period (1 February 2022).

### Statistical Analysis

Statistical analyses were performed using Stata 16 (StataCorp, CollegeStation, Texas, USA). The critical level of statistical significance was set at a p-value less than 0.05. Categorical data were described with frequency and percentage. Normally distributed continuous data was described with mean and standard deviation while non-normally distributed data was described with median and interquartile range. Descriptive analysis was used to describe participants’ characteristics and the prevalence of the comorbidities.

#### External Validation

First, an external validation of the CCI-based prediction model was performed based on the original Charlson study[14]. Ten-year survival probability was calculated as follows: ${10-\text{year survival probability}= 0.983}^{{e}^{(\text{CCI }\times 0.9)}}$. The original CCI model was developed using multivariable Cox regression methods. The model focuses on patients’ comorbidities as predictors, with scores assigned to each predictor. The predictors that are included in the model and scoring system are addressed in Additional file 4, and the equation is shown in Additional file 5. The discriminative ability of the CCI-based model was estimated using Harrell’s C Discrimination Index (or C-statistics). Model calibration was evaluated by visualizing the calibration plot and calculating the calibration slope and the expected observed (E:O) ratio.

#### Model Updating

**Fig. 1:**
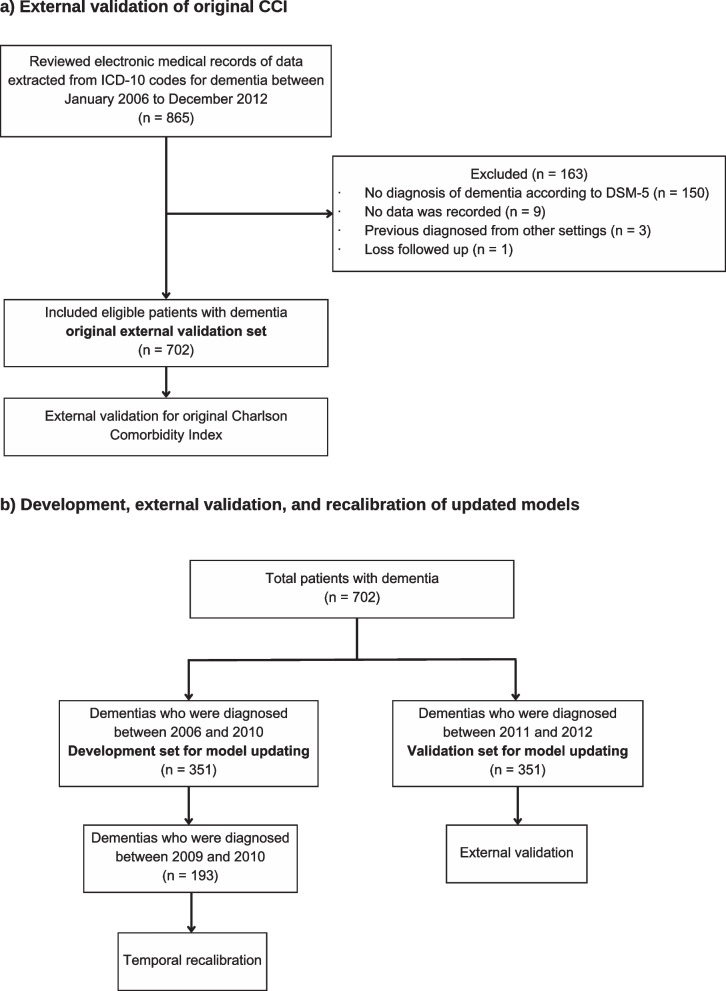
Study Flow Diagram

Model updating was performed with the same cohort since the external performance of the CCI-based model was not satisfactory. The main external validation dataset (n = 702) was temporally split into two datasets to be used during model updating (one dataset (2006 to 2010) for updating (n = 351) and another (2011 to 2012) for validating the updated models (n = 351)). The updated models were developed using the following strategies: 1) re-fitting the model using the original CCI predictors; 2) incorporating new predictors with the original CCI predictors; 3) stepwise backward elimination of predictors from a multivariable analysis. The new predictors were significant associated factors for ten-year survival in patients diagnosed with dementia. These predictors included gender[28–30], health service schemes[17], and co-morbidities out of the CCI, which were hypertension and atrial fibrillation[31]. Gender was included as a binary variable (male or female). The service scheme was included as a categorical variable with four categories (government, self-paid, social, and universal coverage). A multivariable Cox regression was used to determine the predictors for the reduced model using stepwise backward elimination with a statistically significant threshold of a p-value less than 0.100. The details of the predictors included in each model are provided in Additional file 4. The temporal recalibration using the data from 2009 and 2010 was conducted to readjust the baseline survival probability, while all other coefficients in the model remained the same as the derived models. This approach allowed us to correct the survival probabilities, which is a common issue during external validation due to a mismatch in the overall observed event rate and the predicted risk between different cohorts with different diagnosis times[32]. Figure 1 shows a study flow diagram.

## Results

### Patients’ Characteristics

Of 702 patients who were diagnosed with dementia at Maharaj Nakorn Chiang Mai Hospital, the total number of deaths at the end of the follow-up time was 536 (76.35%). Among the patients, the majority were female (56.9%). The average age at the time of dementia diagnosis was 75.22 years old, with a standard deviation of 9.75. The most common types of dementia observed were AD accounting for 39.6% of cases, followed by vascular dementia at 28.8%. The three most prevalent comorbidities among the patients were hypertension at 57.69%, cerebrovascular accident or TIA at 24.93%, and type 2 diabetes mellitus at 21.94%. There was no missing data in the study. Baseline characteristics of participants were described in Table 1.

**Table 1:** Baseline Characteristics of Patients Diagnosed with Dementia

Characteristic	Original external validation set (*N* = 702)		Development set for model updating (*N* = 351)	Validation set for model updating (*N* = 351)	*P*-value
	Missing (%)	Tota*l: n* (%)	Total: *n* (%)	Total: *n* (%)	
Gender					
Male	0	308 (43.87)	166 (47.29)	142 (40.46)	0.068
Female	0	394 (56.913)	185 (52.71)	209 (59.54)	
Age (Mean ± SD)	0	75.22 ± 9.75	72.88 ± 10.32	77.55 ± 8.53	< 0.001
< 50 years	0	11 (1.57)	10 (2.85)	1 (0.28)	< 0.001
50 – 59 years	0	51 (7.26)	39 (11.11)	12 (3.42)	
60 – 69 years	0	107 (15.24)	62 (17.66)	45 (12.82)	
70 – 79 years	0	280 (39.89)	138 (39.32)	142 (40.46)	
≥ 80 years	0	253 (36.04)	102 (29.06)	151 (43.02)	
Type of dementia					
Alzheimer’s disease	0	278 (39.60)	108 (30.77)	170 (48.43)	0.343
Vascular dementia	0	202 (28.77)	130 (37.04)	72 (20.51)	
Mixed type	0	83 (11.82)	43 (12.25)	40 (11.40)	
Others	0	48 (6.84)	20 (5.70)	28 (7.98)	
Unspecified	0	91 (12.96)	50 (14.25)	41 (11.68)	
Service scheme					
Government	0	160 (22.89)	100 (28.65)	60 (17.14)	< 0.001
Self-paid	0	71 (10.16)	44 (12.61)	27 (7.71)	
Social	0	7 (1.00)	4 (1.15)	3 (0.86)	
Universal coverage	0	461 (65.95)	201 (57.59)	260 (74.29)	
Charlson Comorbidity Index					
Myocardial infarction	0	36 (5.13)	19 (5.41)	17 (4.84)	0.732
Congestive heart failure	0	25 (3.56)	11 (3.13)	14 (3.99)	0.541
Peripheral vascular disease	0	24 (3.42)	5 (1.42)	19 (5.41)	0.004
Cerebrovascular accident or TIA	0	175 (24.93)	94 (26.78)	81 (23.08)	0.257
Chronic Obstructive Pulmonary Disease	0	24 (3.42)	13 (3.70)	11 (3.13)	0.678
Connective tissue disease	0	2 (0.28)	2 (0.57)	0	0.157
Peptic ulcer	0	4 (0.57)	3 (0.85)	1 (0.28)	0.316
Liver disease	0	12 (1.71)	4 (1.14)	8 (2.28)	0.244
Mild	0	10 (1.42)	3 (0.85)	7 (1.99)	0.444
Moderate to severe	0	2 (0.28)	1 (0.28)	1 (0.28)	
Type 2 Diabetes Mellitus	0	154 (21.94)	69 (19.66)	85 (24.22)	0.144
Diet control	0	67 (9.54)	29 (8.26)	38 (10.83)	0.003
Uncomplicated	0	69 (9.83)	38 (10.83)	31 (8.83)	
Complicated	0	18 (2.56)	2 (0.57)	16 (4.56)	
Hemiplegia	0	13 (1.85)	8 (2.28)	5 (1.42)	0.401
Moderate to severe CKD	0	40 (5.70)	16 (4.56)	24 (6.84)	0.193
Solid tumor	0	37 (5.27)	28 (7.98)	9 (2.56)	0.001
Leukemia	0	1 (0.14)	1 (0.28)	0	0.317
Lymphoma	0	4 (0.57)	4 (1.14)	0	0.045
AIDS	0	3 (0.43)	3 (0.85)	0	0.083
Associated comorbidity					
Hypertension	0	405 (57.69)	220 (62.68)	185 (52.71)	0.007
Atrial fibrillation	0	18 (2.56)	10 (2.85)	8 (2.28)	0.633

### External Validation

**Fig. 2:**
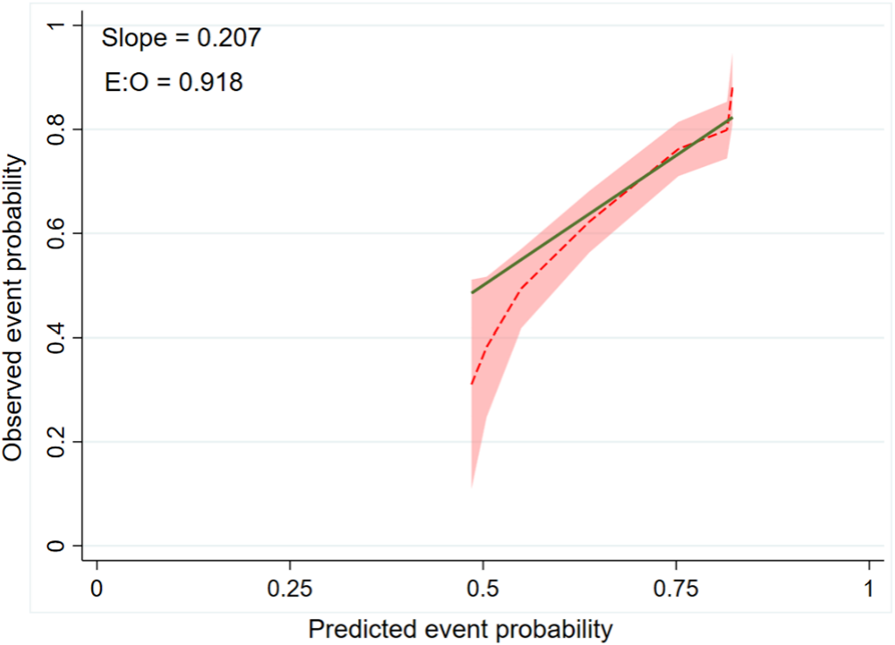
Calibration Plot of External Validation of Original Charlson Comorbidity Index

There exists a disparity between the predicted 10-year survival probability of the original CCI and the observed 10-year survival probability in our dataset. Additional file 6 provides specific numerical values for the survival analysis of the original CCI. The Harrell C-statistics of the CCI-based model was 0.58 (95% CI, 0.54–0.61). The model calibration showed poor external calibration (Fig. 2). The slope of the calibration plot was poor calibration at 0.207, which correlated to an E:O ratio of 0.918 that was underestimated.

### Model Updating

The development and validation of the updated models involved two separate datasets, each consisting of 351 patients. Across both datasets, a majority of the patients were female (52.71% and 59.54%) and a significant portion fell within the age range of 70 to 79 years (39.32% and 40.46%). The most prevalent types of dementia observed were AD at 30.77% and 48.43%, and VD at 37.04% and 20.51% in the respective datasets. Among the patients, the three most common comorbidities were hypertension (62.68% and 52.71%), cerebrovascular accident or TIA (26.78% and 23.08%), and type 2 diabetes mellitus (19.66% and 24.22%) in each dataset, respectively.

Multiple updated models were developed using a development dataset. Model 1 was constructed based on the variables from the original CCI. Model 2 included variables from the CCI as well as additional comorbidity-related factors, including gender, hypertension, atrial fibrillation, and service scheme. Model 3 was derived from Model 2, with stepwise backward elimination. Model 4 encompassed Model 3 and the service scheme. Table 2 presents the adjusted HR with the corresponding p-value obtained from the updated models. Additional file 5 provides detailed instructions on calculating the prognostic index of ten-year survival probability for each model.

**Table 2:** Multivariable Cox Proportional Hazard Regression in Updated Models

	**Model 1**		**Model 2**		**Model 3**		**Model 4**	
**Prognostic factors**	**Adjusted HR** **(95% CI)**	***P*-value**	**Adjusted HR** **(95% CI)**	***P*-value**	**Adjusted HR** **(95% CI)**	***P*-value**	**Adjusted HR** **(95% CI)**	***P*-value**
Gender								
Male			Ref		Ref		Ref	
Female			0.86 (0.66–1.12)	0.277	0.75 (0.59–0.96)	0.025	0.78 (0.61–1.00)	0.050
Age								
< 50 years	Ref		Ref		Ref		Ref	
50 – 59 years	1.94 (0.68–5.55)	0.219	2.61 (0.86–7.94)	0.091	2.04 (0.71–5.91)	0.188	2.49 (0.82–7.55)	0.108
60 – 69 years	2.00 (0.71–5.58)	0.187	3.06 (1.03–9.05)	0.043	2.44 (0.86–6.93)	0.094	3.20 (1.08–9.48)	0.035
70 – 79 years	4.23 (1.57–11.40)	0.004	6.30 (2.17–18.38)	0.001	4.78 (1.73–13.20)	0.003	6.00 (2.05–17.50)	0.001
≥ 80 years	5.08 (1.87–13.83)	0.001	7.61 (2.57–22.54)	< 0.001	5.85 (2.10–16.28)	0.001	7.48 (2.51–22.25)	< 0.001
Service scheme								
Government			Ref				Ref	
Self-paid			0.85 (0.64–1.12)	0.248			0.83 (0.63–1.09)	0.181
Social			1.39 (0.94–2.05)	0.103			1.30 (0.88–1.90)	0.183
Universal coverage			1.56 (0.46–5.25)	0.476			1.35 (0.41–4.45)	0.624
Charlson Comorbidity Index								
Myocardial infarction	1.63 (0.96–2.76)	0.070	1.62 (0.94–2.80)	0.080	1.94 (1.19–3.16)	0.008	1.87 (1.13–3.10)	0.015
Congestive heart failure	1.59 (0.78–3.22)	0.199	1.46 (0.71–2.99)	0.303				
Peripheral vascular disease	0.73 (0.19–2.85)	0.654	0.77 (0.20–3.01)	0.713				
Cerebrovascular accident or TIA	1.01 (0.77–1.36)	0.893	1.02 (0.76–1.37)	0.888				
Chronic Obstructive Pulmonary Disease	1.32 (0.71–2.47)	0.376	1.38 (0.72–2.62)	0.324				
Connective tissue disease	3.08 (0.45–21.08)	0.251	2.83 (0.41–19.46)	0.290				
Peptic ulcer	0.20 (0.03–1.51)	0.119	0.17 (0.02–1.30)	0.087				
Liver disease								
Mild	4.43 (1.30–15.14)	0.018	3.78 (1.09–13.12)	0.036				
Moderate to severe	Omitted	-	Omitted	-				
Type 2 Diabetes Mellitus								
Uncomplicated	1.25 (0.85–1.85)	0.260	1.38 (0.92–2.07)	0.116				
Complicated	1.68 (0.37–7.64)	0.505	1.75 (0.38–8.09)	0.476				
Hemiplegia	2.41 (1.16–4.99)	0.018	2.80 (1.34–5.84)	0.006	2.51 (1.22–5.16)	0.012	2.61 (1.27–5.37)	0.009
Moderate to severe CKD	1.36 (0.78–2.38)	0.284	1.45 (0.82–2.55)	0.199				
Solid tumor	1.42 (0.92–2.19)	0.110	1.30 (0.83–2.04)	0.257				
Leukemia	0.59 (0.08–4.22)	0.597	0.57 (0.08–4.14)	0.576				
Lymphoma	3.07 (1.13–8.32)	0.028	2.90 (1.05–7.97)	0.039	2.55 (0.93–6.98)	0.067	2.63 (0.96–7.19)	0.060
AIDS	7.39 (2.03–26.85)	0.002	7.29 (1.94–27.40)	0.003	7.55 (2.07–27.55)	0.002	7.41 (2.00–27.43)	0.003
Associated comorbidity								
Hypertension			0.87 (0.66–1.14)	0.301				
Atrial fibrillation			1.54 (0.79–3.01)	0.203	1.60 (0.84–3.02)	0.153	1.47 (0.77–2.81)	0.239

Model 1: variables in CCI; Model 2: Model 1 + gender + service scheme + other associated comorbidities; Model 3: Reduced model; Model 4: Model 3 + service scheme

Regarding the discrimination performance of the updated models, the Harrell C-statistics for model validation in the validation set were as follows: 0.60 for Model 1, 0.63 for Model 2, 0.61 for Model 3, and 0.62 for Model 4. These values were slightly lower than the Harrell C-statistics obtained from the development data (Table 3).

**Table 3:** Performance of Original CCI and New Development Models for Survival in Thai Patients Diagnosed with Dementia

Discrimination Measures	**CCI**		**Model 1**		**Model 2**		**Model 3**		**Model 4**	
	C	95% CI	C	95% CI	C	95% CI	C	95% CI	C	95% CI
Development			0.65	0.62–0.69	0.65	0.62–0.69	0.64	0.61–0.68	0.64	0.61–0.68
Validation	0.58*	0.54–0.61	0.60	0.57–0.64	0.63	0.60–0.67	0.61	0.57–0.65	0.62	0.58–0.66
Temporal recalibration			0.60	0.56–0.64	0.63	0.59–0.67	0.61	0.57–0.65	0.61	0.58–0.65

^*^Using external validation set I; Model 1: variables in CCI; Model 2: Model 1 + gender + service scheme + other associated comorbidities; Model 3: Reduced model; Model 4: Model 3 + service scheme

**Fig. 3:**
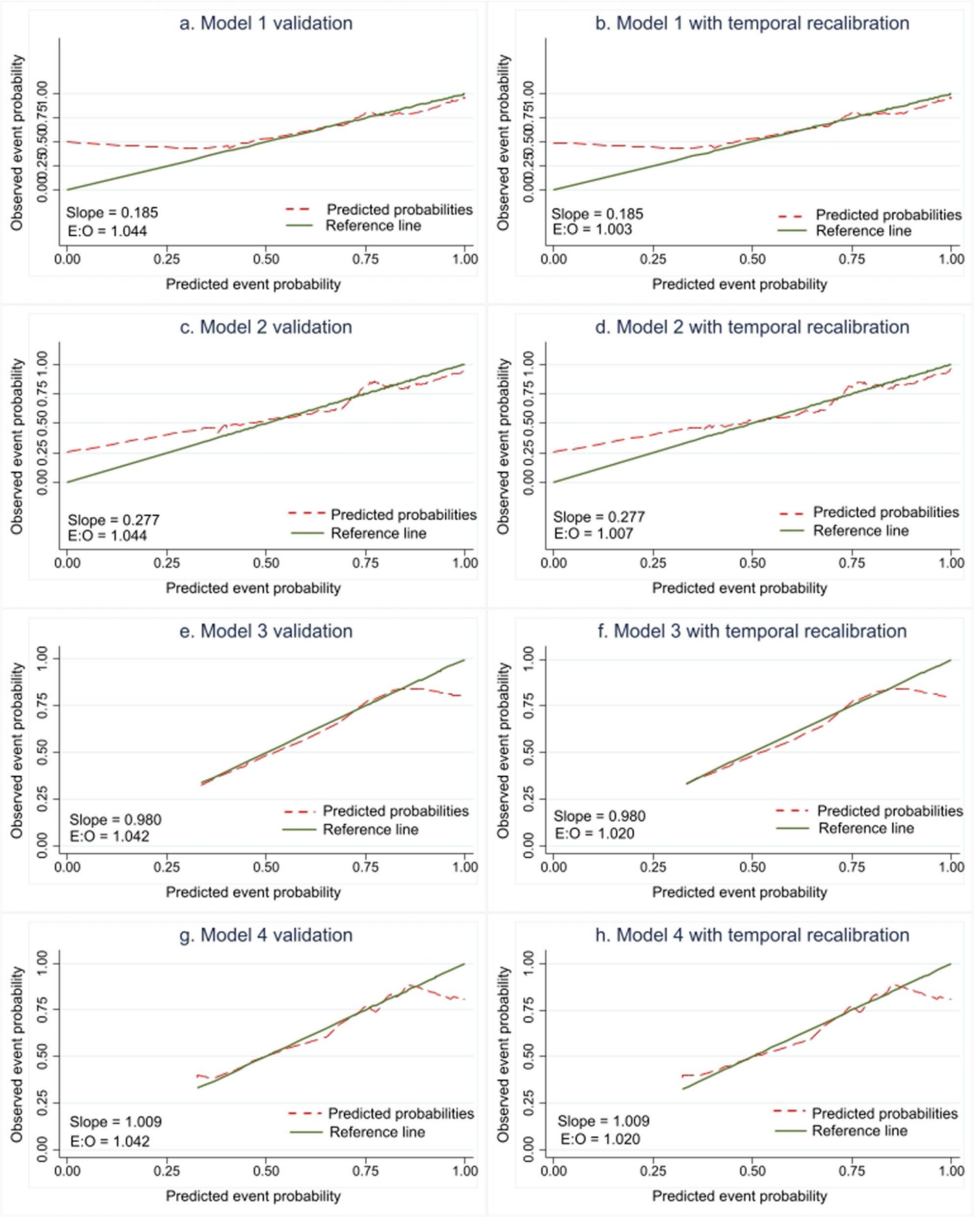
Calibration Plot of Updated Model. Model 1: variables in CCI; Model 2: Model 1 + gender + service scheme + other associated comorbidities; Model 3: Reduced model; Model 4: Model 3 + service scheme

Figure 3 illustrates the calibration plots for model validations, both before and after temporal recalibration. Specifically, Figs. 3a and 3b represent Model 1, Figs. 3c and 3d represent Model 2, Figs. 3e and 3f represent Model 3, and Figs. 3g and 3h represent Model 4. The validation of Models 1 and 2 predicted an underestimation of the probability of death in cases with lower death probabilities. In contrast, Models 3 and 4 appeared to be well calibrated compared to Models 1 and 2, albeit slightly overestimating the probability of death by 0.75 to 1.0 when compared to the actual probability. After recalibrating the models, a slight improvement was observed in the E:O ratio for all models. The time-dependent Area under the ROC Curve (AUROC) for model development, temporal validation, and temporal recalibrated model is illustrated in Additional file 7. The time-dependent AUROCs of nearly all models showed fair discriminative ability across development, temporal validation, and temporal recalibration. However, model 2 performed better in certain cases, demonstrating good discriminative ability with AUROC values of 0.712 and 0.713 at 9 and 10 years during development, and 0.729 and 0.724 at 1 and 2 years during temporal recalibration.

## Discussion

The CCI-based model showed fair performance for survival prediction in Thai patients diagnosed with dementia in terms of discrimination and calibration. Although several models were constructed during updating to identify the optimal set of predictors to be used, the performance of all models was not significantly improved when compared to the initial external performance. Thus, it is doubtful that the CCI-based model is appropriate for predicting survival in this particular domain of patients.

The CCI was initially developed in 1986 and has been widely used as a predictor of one-year mortality in hospitalized patients[14]. However, it is important to recognize that the population included in this study was recruited from an OPD setting, which may introduce variations in the model's predictive accuracy due to differences in settings and circumstances[33]. It is crucial to acknowledge that the CCI was not specifically designed for use in patients diagnosed with dementia and may not always accurately capture the complexity of their condition. Moreover, the CCI focuses on a limited number of comorbid conditions and may not encompass the full range of comorbidities that can impact patients diagnosed with dementia. Therefore, when assessing the health and prognosis of a dementia patient, it is essential to consider the CCI as just one piece of information among many. In a previous study[31], we discovered that among patients diagnosed with dementia, only myocardial infarction, type 2 diabetes mellitus, and liver disease showed a significant association with ten-year mortality, while other comorbidities included in the CCI did not. This highlights the importance of considering additional factors that could potentially influence mortality in these patients, beyond what the CCI encompasses.

It has been established that comorbidities can significantly impact the risk of mortality in patients. In the case of patients diagnosed with dementia, previous research[12, 34–37] has highlighted the influential role of comorbidities in mortality outcomes. This study specifically emphasizes the potential of a reduced model for predicting prognosis in Thai dementia cases. The reduced model incorporates several key factors associated with mortality in patients diagnosed with dementia, including age, gender, myocardial infarction, hemiplegia, lymphoma, AIDS, and atrial fibrillation. Age and gender have consistently emerged as predictors of mortality in individuals with dementia, with increasing age and male gender serving as significant risk factors[38]. Various comorbidities have been identified as contributing to dementia morbidity[39]. Furthermore, prior investigations have established a link between mortality in patients diagnosed with dementia and coronary heart disease, particularly myocardial infarction[37, 40]. In the context of AIDS, the presence of this condition can worsen the risk and severity of infections, which are a leading cause of death in patients diagnosed with dementia [41]. Conversely, cognitive impairment has been found to increase the likelihood of mortality in HIV-positive patients[42]. Another contributing factor to mortality risk is atrial fibrillation, which can result from inadequate use of anticoagulants and a higher incidence of thrombotic events[43]. Consequently, this condition may contribute to a decrease in warfarin prescriptions[44].

Previous research conducted on incident hemodialysis patients in Korea revealed that the majority of comorbidities assessed by the CCI were significant predictors of mortality within this patient population[45]. Moreover, the CCI has demonstrated satisfactory predictive capability for all-cause mortality in hospitalized patients[46, 47], and older adults[48]. In line with these findings, this study aimed to develop a comorbidity-based mortality prediction model specifically tailored for patients with dementia. However, it is important to note that model discrimination, as measured by the C-statistic, is generally considered good when it exceeds 0.7 and superior when it surpasses 0.8[49]. The comorbidity-based survival prediction models employed in this study demonstrated fair discrimination performance, indicating that there are likely other significant factors beyond comorbidities that influence prognosis in dementia. Such factors may include the severity of the disease, socioeconomic status, and caregiver involvement, which must be taken into account[38]. To gain a comprehensive understanding of dementia survival and prognosis, future research should consider investigating additional potential factors and their impact across multiple settings throughout the country. This broader approach will contribute to a more holistic view of dementia outcomes and aid in the development of robust prediction models.

This study demonstrates the potential of comorbidity-based prediction models for mortality prediction in dementia, utilizing readily available data such as age, gender, and comorbidity information that can be obtained from history-taking or medical records. However, it is important to acknowledge the limitations of this study. Firstly, the data used in this study was obtained through retrospective review, which may result in incomplete or missing data. Nonetheless, efforts were made to review data from both electronic medical records and ICD-10 records to mitigate this limitation. Secondly, the characteristics of the patients included in this study may not fully match those of the original CCI development dataset. Consequently, this mismatch may lead to fair prediction performance with poor calibration. Thirdly, there were notable differences in characteristics between the development data and the validation data, including variations in age, PVD, type of DM treatment, solid tumor, lymphoma, and hypertension presence. These differences can potentially impact discrimination performances between the two groups. Fourthly, subgroup analysis for dementia subtypes was not conducted. This decision was based on findings from our previous study, which revealed no significant difference in the mortality rates between Alzheimer’s disease and vascular dementia[31], coupled with limitations in sample size to achieve the power. Nevertheless, our study did not observe any statistically significant difference in the proportions of dementia subtypes between the development and validation cohorts, as illustrated in Table 1. Additionally, dementia can lead to disability or functional impairment, which may impact patient outcomes[50]. However, due to challenges in obtaining this information from medical records and our focus on studying factors associated with mortality in dementia, functional impairment is likely to act as mediators in the association pathway. Therefore, we could not include functional status in the analysis. It is worth noting that patients with dementia at the time of first diagnosis in Thailand are mostly in the mild to moderate stages[51]. Hence, this variable may not significantly affect the results of the study. Lastly, it should be noted that the model was developed using different methods and variables compared to the original CCI, and it was based on data derived from the Northern Thai population. Further validation using future datasets should be conducted to enhance generalizability. Overall, while this study highlights the promise of comorbidity-based prediction models in dementia mortality prediction, these limitations should be considered and further research is needed to validate and refine the models in diverse populations.

## Conclusion

The CCI-based model showed poor predictive performance in Thai patients diagnosed with dementia. Although, there was no difference in discrimination performance between the original CCI and updated models. The use of a developed model may benefit when a prognosis needs to be determined in the ambulatory setting. However, it is important to note that other non-included factors influencing prognosis should also be taken into consideration.

### Supplementary Information

Supplementary materials 1.

Supplementary materials 2.

Supplementary materials 3.

Supplementary materials 4.

Supplementary materials 5.

Supplementary materials 6.

Supplementary materials 7.

## Data Availability

The datasets used and/or analyzed during the current study are available from the corresponding author on reasonable request.

## Abbreviations

AD: Alzheimer’s disease
AIDS: Acquired immunodeficiency syndrome
AUROC: Area under the ROC Curve
CCI: Charlson’s Comorbidities Index
CHF: Congestive heart failure
CI: Confidence interval
CKD: Chronic kidney disease
COPD: Chronic obstructive pulmonary disease
CTD: Connective tissue disease
CVA: Cerebrovascular accident
DLB: Dementia with Lewy bodies
DM: Type 2 diabetes mellitus
DSM: Diagnostic and Statistical Manual of Mental Disorders
FTD: Frontotemporal dementia
ICD-10: International Classification of Diseases-10
MI: Myocardial infarction
PU: Peptic ulcer
PVD: Peripheral vascular disease
SD: Standard deviation
TIA: Transient ischemic attack
TRIPOD: Transparent Reporting of a multivariable prediction model for Individual Prognosis Or Diagnosis
