# Supplementary material for: External Validation of the Charlson Comorbidity Index-based Model for Survival Prediction in Thai Patients Diagnosed with Dementia
Source: BMC Geriatr. 2024 Aug 12;24:675. doi: 10.1186/s12877-024-05238-0 (PMC11318235; doi:10.1186/s12877-024-05238-0)
Supplement: Supplementary file 2 — Supplementary materials 2. [file 12877_2024_5238_MOESM2_ESM.docx]

**Supplementary table 1 ICD-10 codes for diagnosis dementia**

| **ICD-10** | **Disease** |
| --- | --- |
| F00 | Dementia in Alzheimer disease |
| F01 | Vascular dementia |
| F02* | Dementia in other diseases classified elsewhere |
| F03 | Unspecified dementia |
| Other degenerative diseases of the nervous system (G30-G32) | |
| G30 | Alzheimer disease |
| G30.0 | Alzheimer disease with early onset |
| G30.1 | Alzheimer disease with late onset |
| G30.8 | Other Alzheimer disease |
| G30.9 | Alzheimer disease, unspecified |
| G31 | Other degenerative diseases of nervous system, not elsewhere classified |
| G31.0 | Circumscribed brain atrophy |
| G31.1 | Senile degeneration of brain, not elsewhere classified |
| G31.8 | Other specified degenerative diseases of nervous system |
| G31.9 | Degenerative disease of nervous system, unspecified |
| G32* | Other degenerative disorders of nervous system in diseases classified elsewhere |
| G32.8* | Other specified degenerative disorders of nervous system in diseases classified elsewhere |
