# Supplementary material for: External Validation of the Charlson Comorbidity Index-based Model for Survival Prediction in Thai Patients Diagnosed with Dementia
Source: BMC Geriatr. 2024 Aug 12;24:675. doi: 10.1186/s12877-024-05238-0 (PMC11318235; doi:10.1186/s12877-024-05238-0)
Supplement: Supplementary file 3 — Supplementary materials 3. [file 12877_2024_5238_MOESM3_ESM.docx]

**Supplementary table 2 ICD-10 codes for defining comorbidities**

| **Disease** | **ICD-10** |
| --- | --- |
| Hypertension | I10, I11, I12, I13, I15 |
| Atrial fibrillation | I48 |
| Myocardial infarction | I21, I22, I252 |
| Congestive heart failure | I50 |
| Cerebral vascular accident or TIA | I60, I61, I62, I63, I65, I66,G450, G451, G452, G458, G459, G46, I64, G454, I670, I671, I672, I674, I675, I676, I677 I678, I679, I681, I682, I688, I69 |
| Peripheral vascular disease | I71, I790, I739, R02, Z958, Z959 |
| Chronic obstructive pulmonary disease | J40, J41, J42, J44, J43, J45, J46, J47 |
| Peptic ulcer | K25, K26, K27, K28 |
| Liver disease | K702, K703, K73, K717, K740, K742, K746, K743, K744, K745, K729, K766, K767, K721 |
| Moderate to severe CKD | N03, N052, N053, N054, N055, N056, N072, N073, N074, N01, N18, N19, N25 |
| Connective tissue disorder | M32, M34, M332, M053, M058, M059, M060, M063, M069, M050, M052, M051, M353 |
| Parkinson’s disease | G20 |
| Any mental disorder | F20, F31, F32, F33, F34, F38, F39 |
| Type 2 diabetes | E109, E119, E139, E149, E101, E111, E131, E141, E105, E115, E135, E145 |
| Leukemia | C90, C91, C92, C93, C94, C95, C96 |
| Lymphoma | C81, C82, C83, C84, C85, C86, C88 |
| Solid tumor | C00 - C80 |
| AIDS | B20, B21, B22, B23, B24 |
