# Supplementary material for: External Validation of the Charlson Comorbidity Index-based Model for Survival Prediction in Thai Patients Diagnosed with Dementia
Source: BMC Geriatr. 2024 Aug 12;24:675. doi: 10.1186/s12877-024-05238-0 (PMC11318235; doi:10.1186/s12877-024-05238-0)
Supplement: Supplementary file 4 — Supplementary materials 4. [file 12877_2024_5238_MOESM4_ESM.docx]

| **Variable** | **Predictors** | **Detail** | **Input Value** |
| --- | --- | --- | --- |
| AGEGR0 | Patient age at dementia diagnosis (years) | < 50 years (ref.) | AGEGR0 = 1 |
| AGEGR1 |  | 50 – 59 years | AGEGR1 = 1 |
| AGEGR2 |  | 60 – 69 years | AGEGR2 = 1 |
| AGEGR3 |  | 70 – 79 years | AGEGR3 = 1 |
| AGEGR4 |  | ≥ 80 years | AGEGR4 = 1 |
| FEMALE | Patient sex | Male | FEMALE = 0 |
|  |  | Female | FEMALE = 1 |
| MI | Myocardial infraction | No | MI = 0 |
|  |  | Yes | MI = 1 |
| CHF | Congestive heart failure | No | CHF = 0 |
|  |  | Yes | CHF = 1 |
| PVD | Peripheral vascular diseases | No | PVD = 0 |
|  |  | Yes | PVD = 1 |
| CVD | Cerebrovascular diseases | No | CVD = 0 |
|  |  | Yes | CVD = 1 |
| COPD | Chronic obstructive pulmonary disease | No | COPD = 0 |
|  |  | Yes | COPD = 1 |
| CTS | Connective tissue diseases | No | CTS = 0 |
|  |  | Yes | CTS = 1 |
| PU | Peptic ulcer | No | PU = 0 |
|  |  | Yes | PU = 1 |
| DM0 | Diabetes Mellitus status | None or diet-controlled (ref.) | DM0 = 1 |
| DM1 |  | Uncomplicated | DM1 = 1 |
| DM2 |  | Complicated | DM2 = 1 |
| LIVER0 | Liver diseases | None (ref.) | LIVER0 = 1 |
| LIVER1 | - Mild = chronic hepatitis (or cirrhosis without portal hypertension) | Mild | LIVER1 = 1 |
| LIVER3 | - Moderate = cirrhosis and portal hypertension but no variceal bleeding history - Severe = cirrhosis and portal hypertension with variceal bleeding history | Moderate to severe | LIVER3 =1 |
| HEMI | Hemiplegia | No | HEMI = 0 |
|  |  | Yes | HEMI = 1 |
| CKD | Chronic kidney disease   - Moderate = creatinine >3 mg/dL (0.27 mmol/L) - Severe = on dialysis, status post kidney transplant, uremia | None or mild | CKD = 0 |
|  |  | Moderate to severe | CKD = 1 |
| STUMOR | Solid tumors | No | STUMOR = 0 |
|  |  | Yes | STUMOR = 1 |
| M_STUMOR | Metastatic | No | M_STUMOR = 0 |
|  |  | Yes | M_STUMOR = 1 |
| LEUK | Leukemia | No | LEUK = 0 |
|  |  | Yes | LEUK = 1 |
| LYMP | Lymphoma | No | LYMP = 0 |
|  |  | Yes | LYMP = 1 |
| AIDS | Acquired immune-deficiency syndromes | No | AIDS = 0 |
|  |  | Yes | AIDS = 1 |
| HTN | Hypertension | No | HTN = 0 |
|  |  | Yes | HTN = 1 |
| AF | Atrial fibrillation | No | AF = 0 |
|  |  | Yes | AF = 1 |
| HS1 | Health service schemes | Self-paid | HS1 = 1 |
| HS2 |  | Social service scheme | HS2 = 1 |
| HS3 |  | Universal coverage | HS3 = 1 |
| HS4 |  | Government service scheme (ref.) | HS4 =1 |

**Supplementary Table 3** Variable and predictors in the models

**Abbreviations**: ref., reference category.
