# Supplementary material for: External Validation of the Charlson Comorbidity Index-based Model for Survival Prediction in Thai Patients Diagnosed with Dementia
Source: BMC Geriatr. 2024 Aug 12;24:675. doi: 10.1186/s12877-024-05238-0 (PMC11318235; doi:10.1186/s12877-024-05238-0)
Supplement: Supplementary file 5 — Supplementary materials 5. [file 12877_2024_5238_MOESM5_ESM.docx]

**Supplementary Table 4** Calculation the prognostic index of 10-year survival probability by each model

| Baseline 10-year mortality probability (M_10_) | CCI | 0.983 |
| --- | --- | --- |
|  | Model 1 | 0.81 |
|  | Model 2 | 0.82 |
|  | Model 3 | 0.80 |
|  | Model 4 | 0.80 |
| Baseline 10-year mortality probability (M_10_) with temporal recalibration | Model 1 | 0.83 |
|  | Model 2 | 0.83 |
|  | Model 3 | 0.81 |
|  | Model 4 | 0.81 |
| Prognostic index of 10-year mortality probability (PI_10_) | CCI | 0.9 × (AGEGR1 + 2 × AGEGR2 + 3 × AGEGR3 + 4 × AGEGR4 + MI + CHF + PVD + CVD + COPD + CTS + PU + DM1 + 2 × DM2 + LIVER1 + 3 × LIVER3 + 2 × HEMI + 2× CKD + 2 × STUMOR + 6 × M_STUMOR + 2 × LEUK + 2 × LYMP + 6 × AIDS) |
|  | Model 1 | 0.65 × AGEGR1 + 0.77 × AGEGR2 + 1.44 × AGEGR3 + 1.62 × AGEGR4 + 0.48 × MI + 0.47 × CHF – 0.29 × PVD – 0.001 × CVD + 0.28 × COPD + 1.09 × CTS – 1.62 × PU + 0.20 × DM1 + 0.54 × DM2 + 1.42 × LIVER1 – 43.46 × LIVER3 + 0.88 × HEMI + 0.30 × CKD + 0.32 × STUMOR – 0.54 × LEUK + 1.10 × LYMP + 2.00 × AIDS |
|  | Model 2 | 0.96 × AGEGR1 + 1.12 × AGEGR2 + 1.84 × AGEGR3 + 2.03 × AGEGR4 – 0.15 × FEMALE + 0.49 × MI + 0.38 × CHF – 0.26 × PVD + 0.02 × CVD + 0.32 × COPD + 1.04 × CTS – 1.79 × PU + 0.32 × DM1 + 0.56 × DM2 + 1.33 × LIVER1 – 38.56 × LIVER3 + 1.03 × HEMI + 0.37 × CKD + 0.26 × STUMOR – 0.57 × LEUK + 1.06 × LYMP + 1.99 × AIDS – 1.14 × HTN + 0.43 × AF – 0.17 × HS1 + 0.33 × HS2 + 0.44 × HS3 |
|  | Model 3 | 0.70 × AGEGR1 + 0.95 × AGEGR2 + 1.56 × AGEGR3 + 1.75 × AGEGR4 – 0.26 × FEMALE + 0.66 × MI + 0.91 × HEMI + 0.94 × LYMP + 2.02 × AIDS + 0.46 × AF |
|  | Model 4 | 0.91 × AGEGR1 + 1.16 × AGEGR2 + 1.79 × AGEGR3 + 2.01 × AGEGR4 – 0.25 × FEMALE + 0.63 × MI + 0.96 × HEMI + 0.97 × LYMP + 2.00 × AIDS + 0.39 × AF – 0.19 × HS1 + 0.26 × HS2 + 0.30 × HS3 |
| Overall survival probability  at 10-year | | 1 – (M_10_ ^exp(PI^10^)^) |

Variable abbreviations are shown in Supplementary Table 3.
