# Supplementary figures and images for: External Validation of the Charlson Comorbidity Index-based Model for Survival Prediction in Thai Patients Diagnosed with Dementia
Source: BMC Geriatr. 2024 Aug 12;24:675. doi: 10.1186/s12877-024-05238-0 (PMC11318235; doi:10.1186/s12877-024-05238-0)

**Supplementary Figure 1** Numerical values for the survival analysis of the original CCI.


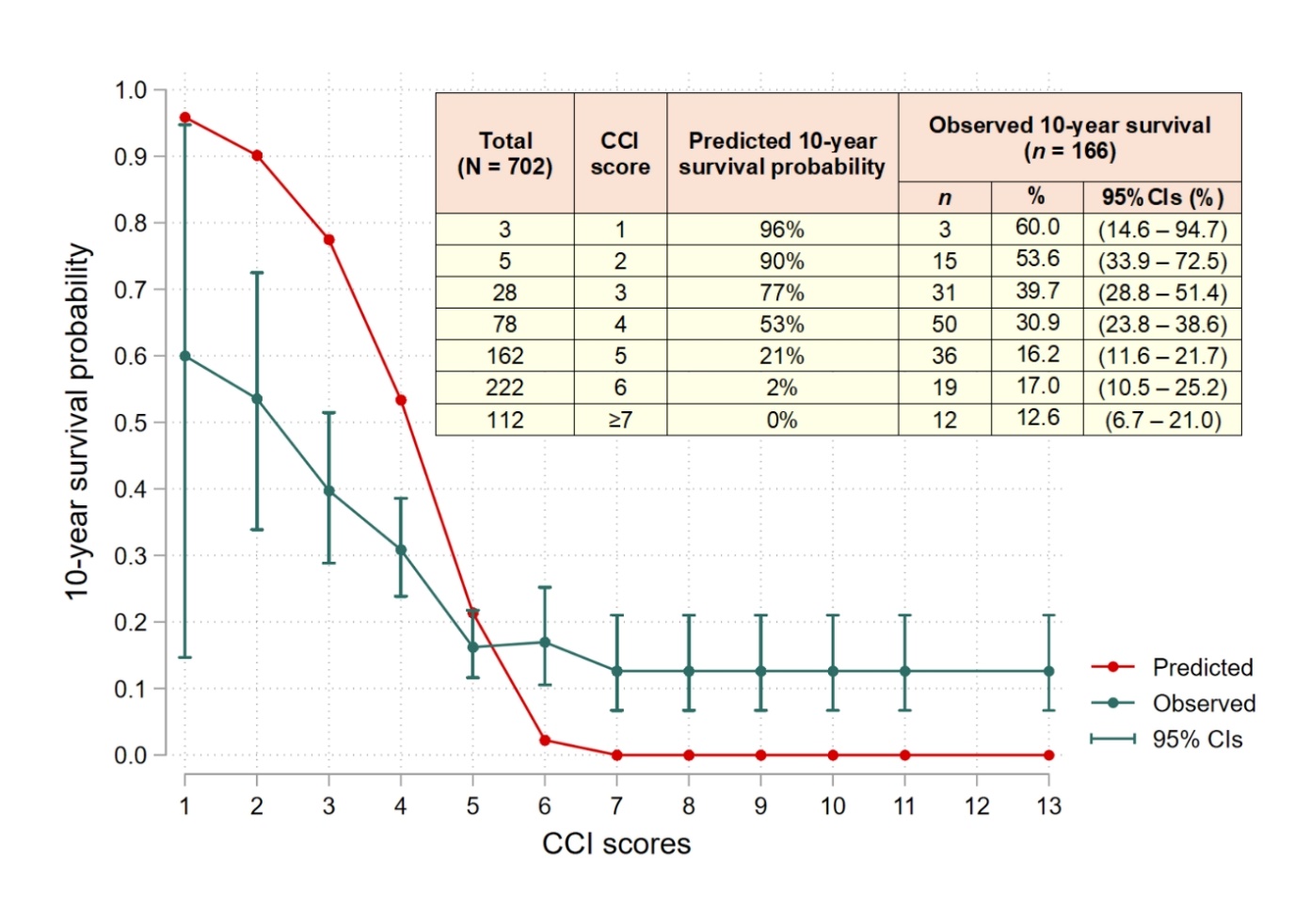

Supplement: Supplementary file 6 — Supplementary materials 6. [file 12877_2024_5238_MOESM6_ESM.docx]
