# Supplementary material for: External Validation of the Charlson Comorbidity Index-based Model for Survival Prediction in Thai Patients Diagnosed with Dementia
Source: BMC Geriatr. 2024 Aug 12;24:675. doi: 10.1186/s12877-024-05238-0 (PMC11318235; doi:10.1186/s12877-024-05238-0)

**Supplementary Figure 2** Time-dependent AUC of all models’ development.


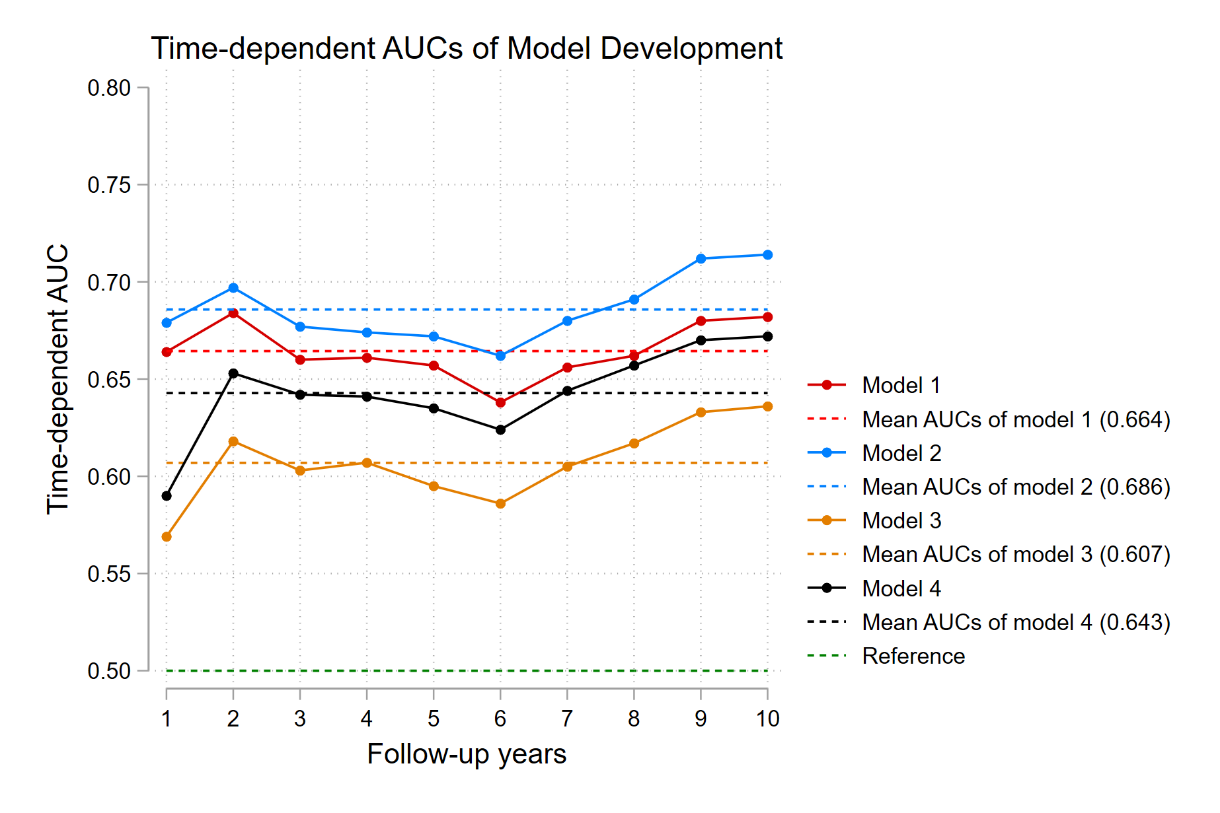


**Supplementary Figure 3** Time-dependent AUC of all models’ temporal validation.


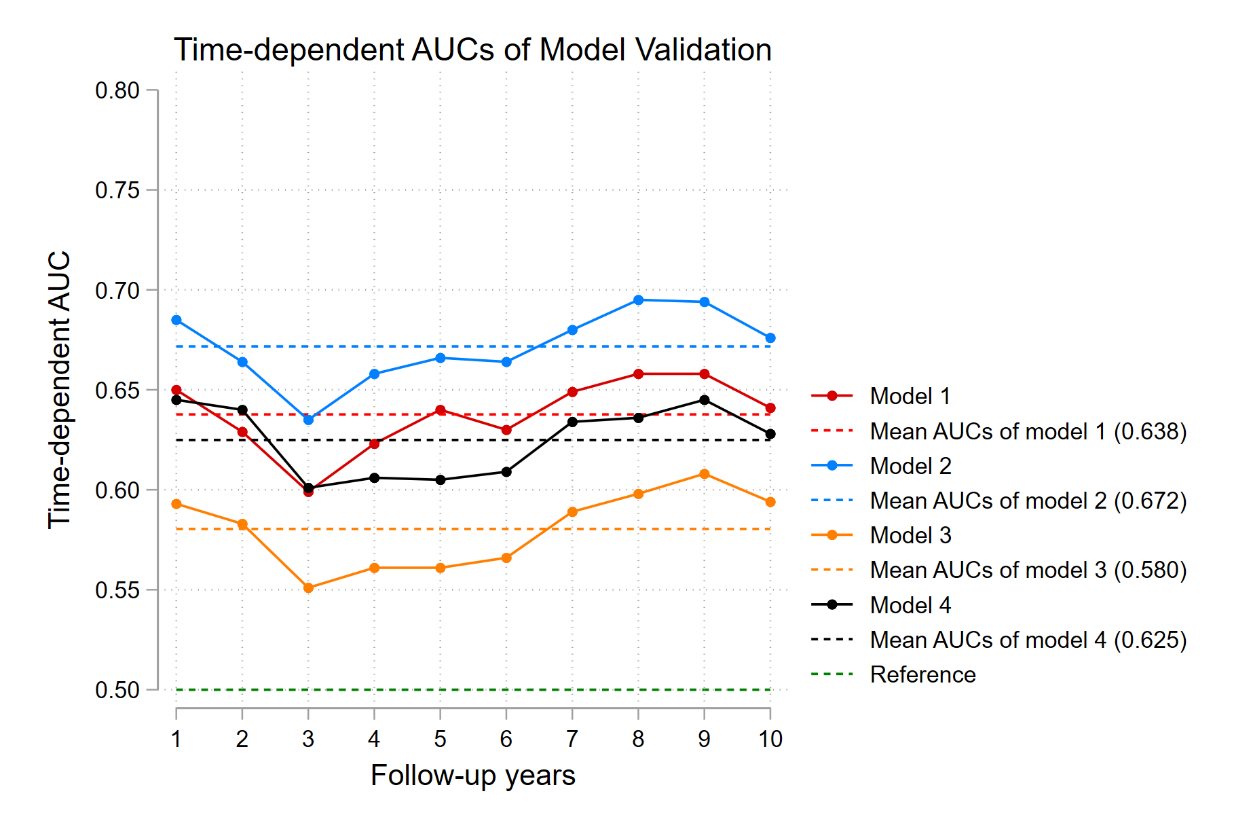


**Supplementary Figure 4** Time-dependent AUC of all temporal recalibrated models’ validation.


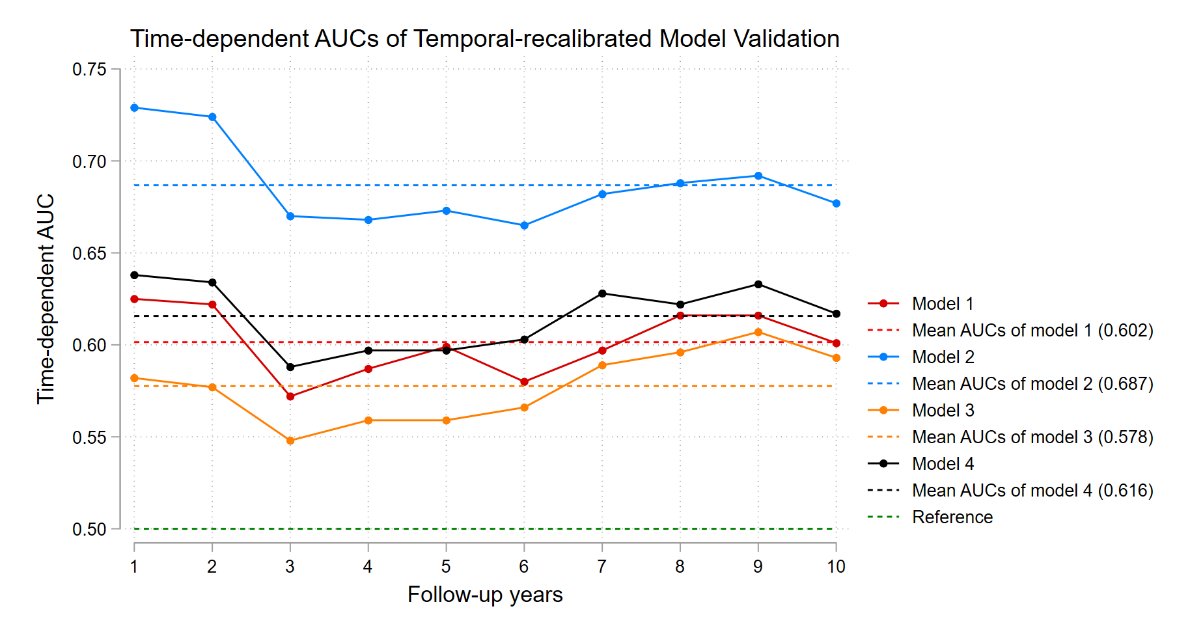

Supplement: Supplementary file 7 — Supplementary materials 7. [file 12877_2024_5238_MOESM7_ESM.docx]
